# Extracellular vesicles from Li-Qi-Yang-Yin formula attenuate LPS-stimulated inflammatory injury in human colonic epithelial cells *in vitro* by delivering bioactive metabolites and suppressing the oxidative stress–inflammation axis

**DOI:** 10.3389/fmed.2026.1751807

**Published:** 2026-03-09

**Authors:** Xiaoyue Deng, Weichen Zhang, Lianjie Xu, Shun Seng Ong, Yingzheng Zhang, Tunyu Jian, Tianshu Xu

**Affiliations:** 1 Department of Traditional Chinese Medicine, Nanjing Drum Tower Hospital, The Drum Tower Clinical Medical College, Nanjing University of Chinese Medicine, Nanjing, China; 2 School of Pharmacy, Nanjing University of Chinese Medicine, Nanjing, China; 3 Jiangsu Key Laboratory for Conservation and Utilization of Plant Sciences, Institute of Botany, Jiangsu Province and Chinese Academy of Sciences, Nanjing, China; 4 Jiangyin Hospital Affiliated to Nanjing University of Chinese Medicine, Jiangyin, China; 5 Department of Traditional Chinese Medicine, Nanjing Drum Tower Hospital, Affiliated Hospital of Medical School, Nanjing University, Nanjing, China

**Keywords:** extracellular vesicles (EVs), inflammatory response, metabolomics, plant-derived extracellular vesicles (PDEV), traditional Chinese medicine formula

## Abstract

**Background:**

The Li-Qi-Yang-Yin (LQYY) formula, a traditional Chinese medicine (TCMs) compound, holds potential in managing gastrointestinal disorders. Extracellular vesicles (EVs) derived from medicinal plants are emerging as novel carriers of bioactive compounds, yet their role in multi-herb formulations like LQYY remains largely unexplored. This study aimed to isolate LEVs from the LQYY formula and investigate their protective effects against inflammation and oxidative stress in intestinal epithelial cells.

**Methods:**

Li-Qi-Yang-Yin formula contains EVs were isolated from the LQYY decoction via ultracentrifugation. Their physicochemical characteristics were characterized using nanoparticle tracking analysis (NTA) and transmission electron microscopy (TEM). The metabolite profile was analyzed by untargeted metabolomics. Using a lipopolysaccharide (LPS)-stimulated inflammatory model in human NCM460 colon epithelial cells, we assessed nitric oxide (NO) production, inflammatory cytokine levels, and the expression of proteins in the Toll-like receptor 4 (TLR4)/nuclear factor-kappa B (NF-κB) pathway.

**Results:**

We successfully isolated LEVs with a typical cup-shaped morphology and an average size of 201.1 nm. Metabolomic analysis identified 862 metabolites, with significant enrichment in fatty acids, phenylpropanoids, and flavonoids. These LEVs were efficiently internalized by NCM460 cells. Pretreatment with LEVs, particularly at a high dose (50 μg/mL), significantly attenuated LPS-stimulated cytotoxicity and intracellular NO overproduction. Furthermore, LEVs dose-dependently suppressed the secretion of pro-inflammatory cytokines (IL-6, TNF-α), promoted the anti-inflammatory cytokine IL-10, and inhibited the activation of the TLR4/NF-κB signaling pathway.

**Conclusion:**

Our findings demonstrate that LEVs from the LQYY formula are natural nanoparticles enriched with anti-inflammatory and antioxidant metabolites and can alleviate LPS-stimulated inflammatory injury in human colonic epithelial cells *in vitro*. These results provide proof-of-concept evidence and warrant further validation in animal models to determine disease relevance.

## Introduction

The intestinal epithelium serves as a critical barrier against luminal pathogens and toxins, and its integrity is essential for maintaining overall gut homeostasis (1). Disruption of this barrier, often driven by excessive oxidative stress and chronic inflammation, is a hallmark of various gastrointestinal diseases, including inflammatory bowel disease (IBD) (2). Lipopolysaccharide (LPS), a major component of the outer membrane of Gram-negative bacteria, is a potent inflammatory stimulus that triggers the release of pro-inflammatory cytokines and reactive oxygen species (ROS) primarily through activation of the Toll-like receptor 4 (TLR4)/nuclear factor-kappa B (NF-κB) pathway, thereby playing a key role in initiating and perpetuating intestinal inflammation (3–5).

The Li-Qi-Yang-Yin (LQYY) formula was developed based on the classical prescription Zengye Tang. Zengye Tang (Fluid-Increasing Decoction) is a classical three-herb prescription first recorded in Wu Jutong’s Wenbing Tiaobian (Treatise on Differentiation and Treatment of Warm Diseases, Qing dynasty). It consists of *Scrophulariae Radix* (Xuanshen), *Ophiopogonis Radix* (Maidong), and *Rehmanniae Radix* (Dihuang), and has been traditionally used to nourish yin, generate fluids, and moisten intestinal dryness, particularly for constipation described as “dry bound stool” associated with yin-fluid deficiency (6). In contemporary practice, LQYY was empirically developed on the basis of this core therapeutic principle by incorporating additional herbs aimed at facilitating qi movement and enhancing intestinal lubrication to better match modern clinical presentations. It has been used clinically as an empirical remedy to improve intestinal lubrication and relieve constipation, while we also hypothesize that this may be achieved by modulating intestinal dysbiosis, barrier dysfunction, and inflammatory responses. Its composition includes the following herbs: *Adenophorae Radix* (Nanshashen, 20 g), *Ophiopogonis Radix* (Maidong, 20 g), *Scrophulariae Radix* (Xuanshen, 30 g), *Rehmanniae Radix* (Shudihuang, 30 g), *Armeniacae Semen Amarum* (Xingren, 10 g), *Atractylodis Macrocephalae Rhizoma* (Baizhu, 40 g), *Aurantii Fructus* (Zhiqiao, 15 g), *Magnoliae Officinalis* Cortex (Houpo, 10 g), *Trichosanthis Semen* (Gualouren, 20 g), *Cannabis Fructus* (Huomaren, 10 g), *Pruni Semen* (Yuliren, 15 g), and *Aucklandiae Radix* (Muxiang, 8 g). Despite its documented clinical use, the specific bioactive constituents and their mechanisms of action remain poorly characterized, significantly limiting the broader therapeutic application of this formula. Further investigation is therefore essential to elucidate its pharmacodynamic foundation and underlying molecular mechanisms.

Recently, plant-derived extracellular vesicles (EVs) have emerged as a novel class of natural nanoparticles. These EVs are abundantly present in medicinal plants and have been shown to encapsulate various bioactive metabolites (7). They exhibit exceptional stability, biocompatibility, and the ability to mediate intercellular communication, functioning as efficient delivery vehicles for their natural cargo (8, 9). Despite promising findings from single herbs, research on EVs derived from complex traditional Chinese medicine (TCMs) formulations, which represent the mainstream form of TCMs application, is exceedingly rare. The potential role of EVs as key functional vectors in multi-herb formulations like LQYY is virtually unknown. We hypothesized that the LQYY formula contains EVs (LEVs) that serve as natural carriers of anti-inflammatory and antioxidant compounds, contributing to the formula’s therapeutic effects.

Therefore, the present study was designed to isolate and characterize LEVs from the aqueous extract of the LQYY formula. We further aimed to investigate their biological activities using an *in vitro* model of LPS-stimulated inflammation in human colonic epithelial cells (NCM460). Specifically, we assessed the cellular uptake of LEVs, their effects on nitric oxide (NO) production, inflammatory cytokine secretion, and the regulation of the TLR4/NF-κB pathway to elucidate their potential protective mechanisms against oxidative stress and inflammation.

## Materials and methods

### Preparation and sucrose density-gradient purification of LEVs

The LQYY formula was obtained from the Jiangyin Hospital of Traditional Chinese Medicine (Wuxi, China). To isolate LEVs, the formula was subjected to aqueous extraction using a water-to-herb ratio of 1:3 (w/v) at 100 °C for 1 h, and this process was repeated twice. The combined extracts yielded approximately 400 mL of solution. This crude extract was sequentially centrifuged at 3,000 × *g* for 30 min, 6,000 × *g* for 30 min, and 10,000 × *g* for 30 min, all at 4 °C, to remove large debris and aggregates. The supernatant was filtered through a 0.45 μm membrane filter and then ultracentrifuged at 100,000 × *g* for 2 h using a Thermo Scientific Sorvall WX100 + ultracentrifuge equipped with a SureSpin 632 rotor to obtain a crude LEVs pellet.

**FIGURE 1:**
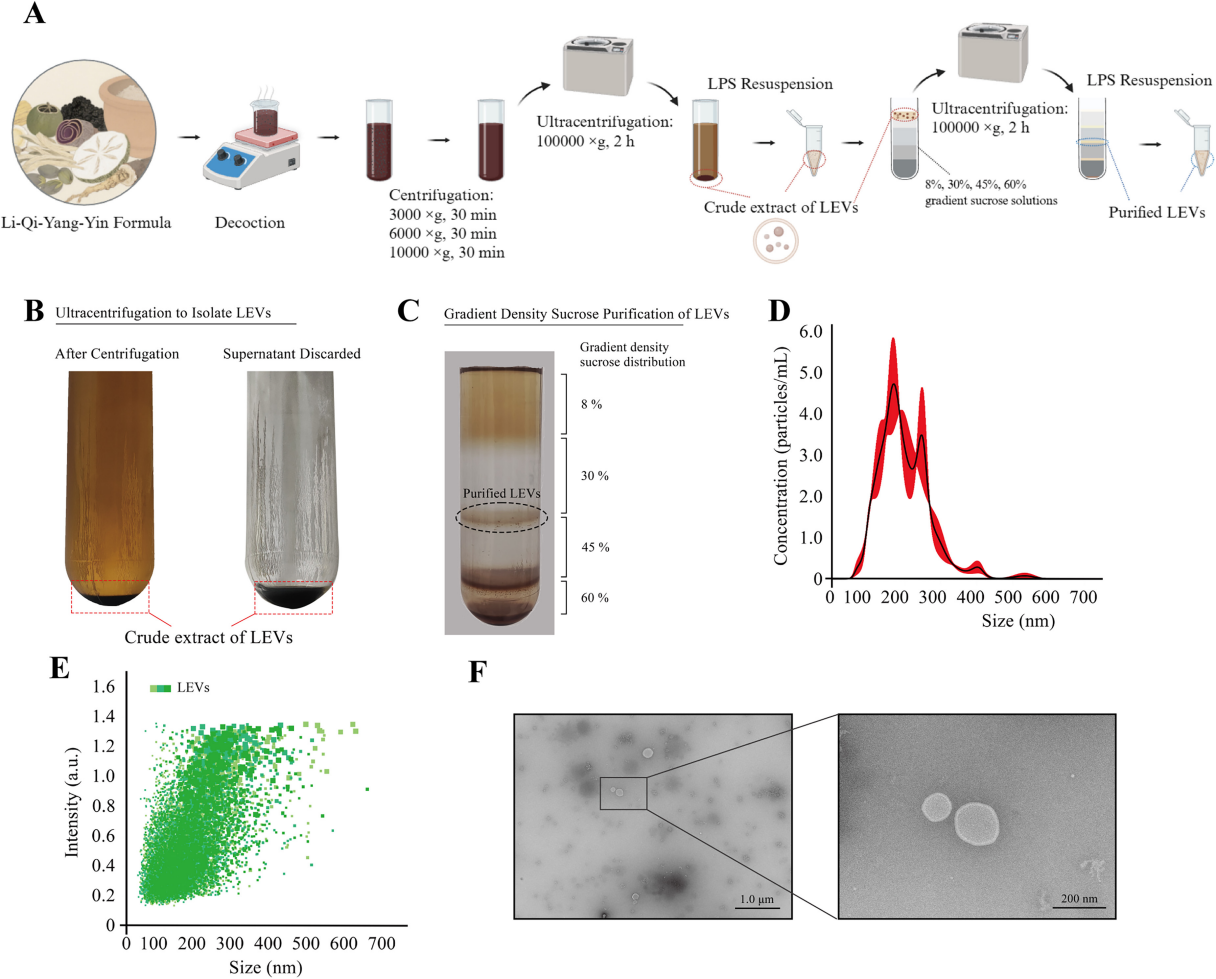
Isolation, sucrose density-gradient purification, and characterization of LEVs derived from the LQYY decoction. **(A)** Workflow for LEVs isolation by differential centrifugation/ultracentrifugation followed by discontinuous sucrose density-gradient ultracentrifugation (DGUC). **(B)** Representative images showing the crude LEVs pellet after ultracentrifugation. **(C)** Discontinuous sucrose step gradient (8%, 30%, 45%, 60%); the vesicle-enriched band at the 30%/45% interface was collected as purified LEVs. **(D,E)** Size distribution and particle concentration measured by NTA. **(F)** Representative TEM images of purified LEVs. TEM images are representative of observations from at least three independent LEV preparations and multiple fields of view. NTA measurements were performed on three independent LEVs preparations, and representative TEM images are shown.

To further remove co-isolated non-vesicular components from the complex herbal decoction, the crude LEVs pellet was purified by discontinuous sucrose density-gradient ultracentrifugation (DGUC). Briefly, sucrose solutions (8%, 30%, 45%, and 60% [w/v]) were carefully layered to form a step gradient. The crude LEVs suspension was loaded onto the gradient and ultracentrifuged at 100,000 × *g* for 2 h at 4 °C. The vesicle-enriched band at the 30%/45% interface was collected, diluted with phosphate-buffered saline (PBS), and ultracentrifuged again at 100,000 × *g* for 2 h at 4 °C to remove sucrose. The resulting pellet was washed once with PBS, resuspended in PBS, aliquoted, and stored at −80 °C until use (Figure 1A).

### LEV quantification and dosing

Given that LEVs isolated from the herbal decoction contained very low detectable protein compared with vesicles derived from fresh plant materials, protein-based normalization was not used for dosing. Instead, LEV concentrations in this study were defined based on lyophilized dry mass. Briefly, an aliquot of the final purified LEV suspension (after DGUC collection and PBS washing) with a known volume was transferred into a pre-weighed low-binding tube, freeze-dried to constant weight, and weighed using an analytical balance. The dry mass concentration (μg/mL) was calculated as: (dry mass, μg)/(aliquot volume, mL).

### Characterization and untargeted metabolomics analysis of LEVs

Purified LEVs were characterized by negative-staining TEM. Briefly, LEV suspensions were diluted in PBS to an appropriate particle density and 5–10 μL was applied onto carbon-coated copper grids that had been glow-discharged immediately before use. After 1–2 min adsorption, excess liquid was blotted with filter paper and the grids were gently rinsed with distilled water. The samples were then negatively stained with 2% (w/v) uranyl acetate for 30 s, blotted, and air-dried at room temperature. Grids were examined using a transmission electron microscope (TEM HT-7700, Hitachi, Japan), and images were acquired. For each independent LEV preparation, multiple grid areas and fields of view were examined. Representative images shown in the manuscript were selected based on typical vesicle morphology observed across fields, and all micrographs include scale bars. Particle size distribution was analyzed with a NanoFCM Flow NanoAnalyzer (N30E; NanoFCM Inc., Xiamen, China). For metabolomic profiling, LEVs were homogenized in 80% methanol, centrifuged to collect supernatants, and vacuum-dried. The resulting residue was reconstituted in 100 μL of 80% methanol prior to liquid chromatography–mass spectrometry (LC–MS) analysis. Chromatographic separation was performed using an UltiMate 3000 UPLC system (Thermo Fisher Scientific, Germany), and metabolites were subsequently detected with a Q-Exactive high-resolution tandem mass spectrometer (Thermo Fisher Scientific, Germany).

### Cell culture

The NCM460 cell line (human normal colon epithelial cells) was procured from SUNNCELL (SNL-519, China). Cells were maintained in RPMI-1640 medium supplemented with 20% (v/v) fetal bovine serum, 100 U/mL penicillin, and 100 U/mL streptomycin, under a humidified atmosphere of 5% CO_2_ at 37 °C.

### Cellular uptake of LEVs

DiO-labeled LEVs were prepared by incubating LEVs with DiO at 37 °C for 30 min, followed by centrifugation at 100,000 × *g* for 2 h to remove any unbound dye. The labeled LEVs were then resuspended in RPMI-1640 medium. For cellular uptake experiments, NCM460 cells were incubated with DiO-labeled LEVs for periods ranging from 0 to 6 h at 37 °C. After incubation, the cells were rinsed thoroughly with PBS to eliminate non-internalized particles. Internalized fluorescence signals were visualized and captured using a Carl Zeiss LSM 900 confocal microscope. Image acquisition and quantitative analysis were performed using ZEN software (Carl Zeiss Microscopy, version 2011). Given the known limitations of lipophilic dye labeling for EVs uptake studies, we interpret DiO signals as indicative of LEVs–cell association and putative internalization, while acknowledging that dye-only controls and orthogonal labeling strategies are required for definitive validation.

### Cell viability assay

The viability of NCM460 cells was determined using a CCK-8 assay kit (BS350C, Biosharp, China). Briefly, cells were seeded into 96-well plates and allowed to adhere. Following attachment, cells were treated with LEVs for 24 h and LPS (HY-D1056, MedChemExpress, USA) for 12 h, respectively. Subsequently, CCK-8 reagent was added to each well and incubated for 4 h at 37 °C. Cell viability was measured spectrophotometrically and calculated in accordance with the manufacturer’s instructions.

### Antioxidant and anti-inflammatory activity assays of LEVs

Cells were plated in 96-well plates. After cell attachment, the LEVs-L and LEVs-H groups were treated with LEVs at concentrations of 25 and 50 μg/mL, respectively, suspended in fresh medium. The untreated control group (CON) and LPS groups received fresh medium only and were pre-incubated for 12 h. Subsequently, the LPS, LEVs-L, and LEVs-H groups were co-treated with LPS at a final concentration of 10 μg/mL for 12 h. After incubation, both cell culture medium and cells were collected. The cells were lysed to prepare cell lysates. Intracellular levels of NO (A013-2-1, Jiancheng Bioengineering Institute, China), Interleukin-6 (IL-6, EK106, Multi Sciences, China), IL-10 (EK110, Multi Sciences, China), and tumor necrosis factor-α (TNF-α, EK182, Multi Sciences, China), as well as extracellular NO levels in the medium, were assayed using NO assay kits and ELISA kits for each cytokine, respectively, in accordance with the manufacturer’s protocols.

To evaluate whether soluble, non-vesicular components co-isolated during LEV preparation contributed to the observed bioactivities, we collected the post-ultracentrifugation supernatant after pelleting crude LEVs (EV-depleted fraction). The supernatant was lyophilized and reconstituted in PBS to obtain a concentration normalized to the same starting decoction volume and adjusted to match the equivalent mass dose used for LEVs-H. This preparation was termed LEVs-Free (LEVs-F). To exclude potential cytotoxic effects of the soluble fraction at the selected dose, NCM460 cells were incubated with LEVs-F at the indicated concentrations, and cell viability was assessed using the CCK-8 assay. Based on these results, an equivalent dose of LEVs-H (50 μg/mL) was selected for the same conditioning and LPS challenge protocol applied to NCM460 cells as for LEVs.

### Western blot

Total protein was extracted from treated NCM460 cells using RIPA lysis buffer supplemented with 1% protease and phosphatase inhibitor cocktail. Protein concentration was determined using the BCA assay according to the manufacturer’s instructions. Extracted proteins were separated by electrophoresis on 10% SDS-PAGE gels and then transferred to PVDF membranes. Membranes were blocked with 5% non-fat dry milk for 2 h at room temperature. Then, they were incubated overnight at 4 °C with the following primary antibodies: p-NF-κB (1:2000, 82335-1-RR, Proteintech, China), NF-κB (1:2000, 10745-1-AP, Proteintech, China), TLR4 (1:2000, 19811-1-AP, Proteintech, China), and β-actin (1:40000, 66009-1-Ig, Proteintech, China). Following washing, membranes were incubated with horseradish peroxidase (HRP)-conjugated secondary antibodies (1:5000, anti-rabbit: R00001, anti-mouse: M00001, Nature Biosciences, China) for 2 h at room temperature. Protein bands were visualized using an enhanced chemiluminescence (ECL, BL520B, Biosharp, China) detection kit and captured with a chemiluminescence imaging system. Quantification was performed using ImageJ 1.54p.

### Statistical analysis

All quantitative data are presented as mean ± standard deviation (SD). The value of n indicates the number of independent biological replicates, as specified in the corresponding figure legends. For comparisons among multiple groups, one-way analysis of variance (ANOVA) followed by Tukey’s multiple-comparison *post-hoc* test was applied. Statistical analyses were performed using GraphPad Prism 10.1.2. *P*-value ≤ 0.05 was considered statistically significant.

## Results

### Isolation and characterization of LEVs

Because the LQYY formula is typically administered orally, we prepared the LQYY decoction using a standard traditional Chinese medicine decoction procedure (decocted twice and combined) (10, 11). The decoction was subjected to sequential low-speed centrifugation, filtration, and ultracentrifugation to obtain a crude LEVs pellet (Figures 1A,B).

Given the complexity of herbal extracts and the risk of co-isolating non-vesicular macromolecular/colloidal components, we further purified the crude pellet using discontinuous sucrose DGUC (8%, 30%, 45%, and 60% sucrose) (12–14). A distinct pale yellowish-brown band was consistently observed at the 30%/45% interface and was collected as purified LEVs (Figure 1C). After dilution and washing to remove sucrose, nanoparticle tracking analysis (NTA) showed that LEVs had a mean diameter of 201.1 ± 72.7 nm (Figures 1D,E). Notably, when the LEVs stock had a lyophilized dry-mass concentration of ∼142 μg/mL, the corresponding particle concentration measured by NTA was (6.57 ± 0.28) × 10^9^ particles/mL. Transmission electron microscopy (TEM) revealed vesicles with typical cup-shaped morphology and intact membrane structures (Figure 1F), supporting successful enrichment of extracellular vesicles (15). Throughout the manuscript, “LEVs” refers to this DGUC-purified vesicle fraction operationally defined by the isolation procedure and size distribution, without inferring a specific biogenesis pathway (e.g., “exosomes”).

### LEVs harbor a diverse array of bioactive metabolites

**FIGURE 2:**
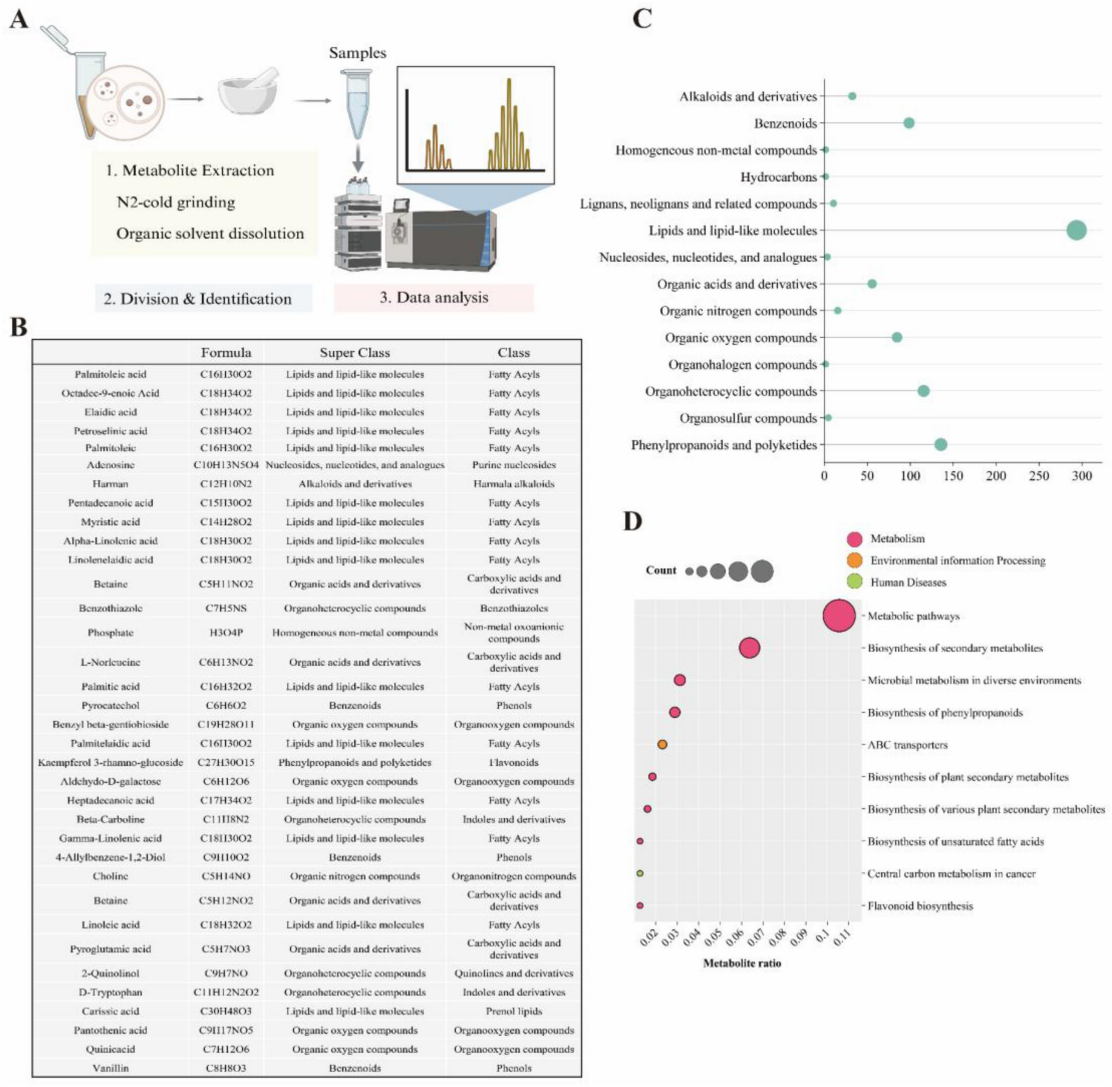
Metabolomic profiling of LEVs. **(A)** Workflow for metabolite extraction from purified LEVs and subsequent identification via liquid chromatography–mass spectrometry (LC–MS) (*n* = 6). **(B)** Composition of metabolites identified in LEVs, presented based on database matching confidence (score = 1). **(C)** Functional annotation of metabolites using the Human Metabolome Database (HMDB). **(D)** Bubble chart summarizing Kyoto Encyclopedia of Genes and Genomes (KEGG) pathway enrichment analysis.

Given that the LQYY formula comprises numerous TCMs, which have been shown to exert biological activities via various metabolites, we conducted a metabolomic analysis of LEVs to identify potential active components and infer their functions (Figure 2A). A total of 862 metabolites were identified in LEVs. Figure 2B displays the top 35 metabolites with high database matching scores, which encompass diverse classes including lipids and lipid-like molecules, nucleosides, nucleotides and analogues, alkaloids and derivatives, as well as organic acids and derivatives. Notably, fatty acids were abundantly detected (Figure 2C). KEGG pathway analysis revealed significant enrichment of metabolites in pathways related to metabolism pathways, biosynthesis of secondary metabolites, biosynthesis of phenylpropanoids, flavonoid biosynthesis, and biosynthesis of unsaturated fatty acid–pathways often associated with the primary active constituents of TCMs. Interestingly, under the human disease category, central carbon metabolism in cancer was enriched, implying that LEVs contain numerous potential bioactive components that may contribute to their efficacy in humans (Figure 2D).

### LEVs were internalized by NCM460 cells

**FIGURE 3:**
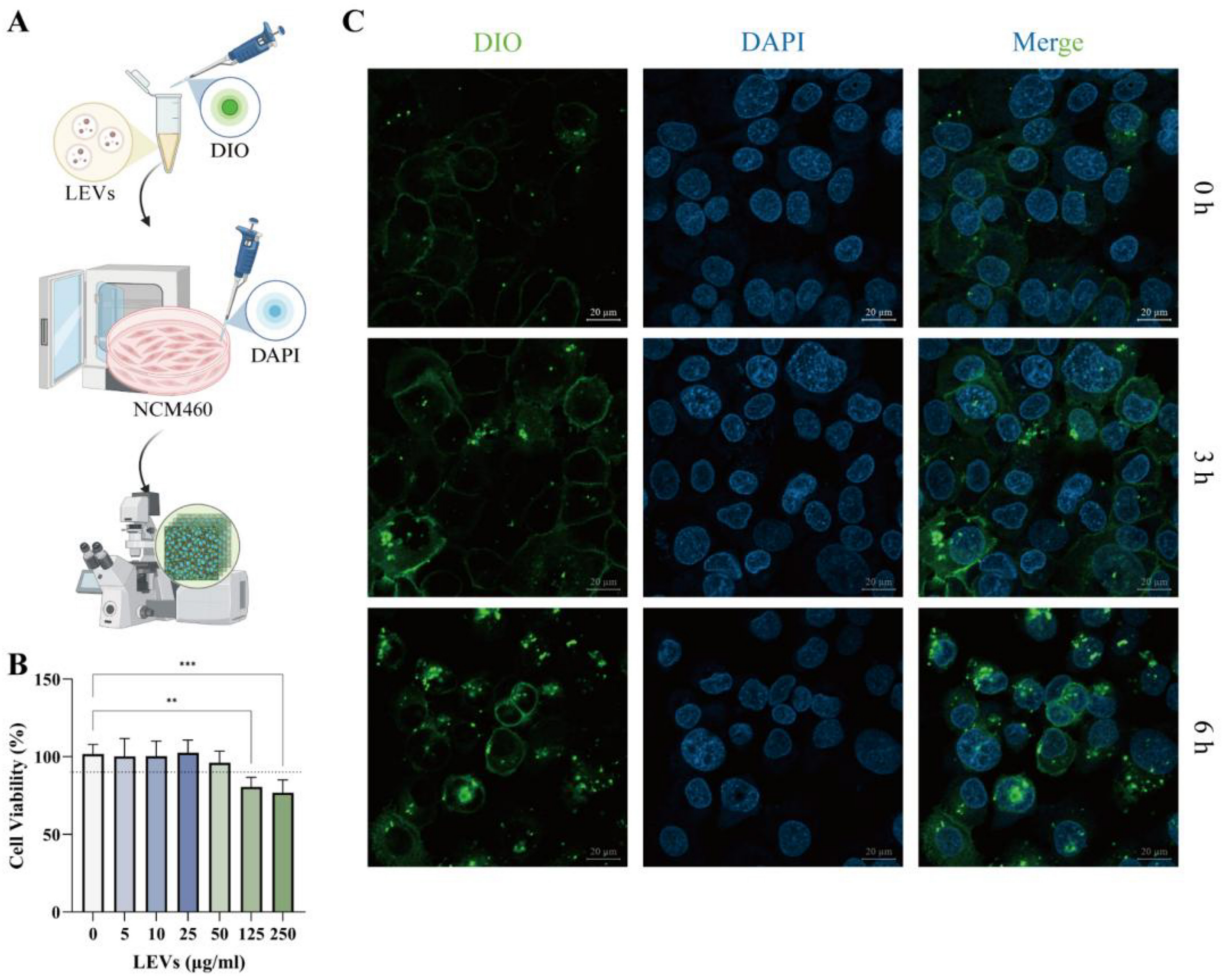
Internalization of LEVs in NCM460. **(A)** Internalization of DiO-labeled LEVs (green) in NCM460 cells counterstained with DAPI (blue) was assessed following co-culture. **(B)** Cell viability was evaluated after 24-h exposure to LEVs at concentrations ranging from 0 to 250 μg/mL. **(C)** Representative fluorescence microscopy images illustrating the time-dependent uptake of DiO-labeled LEVs (green) by NCM460 cells at 0, 3, and 6 h. Nuclei are counterstained with DAPI (blue). Data were expressed as mean ± SD, **P < 0.01 and ***P < 0.001 (*n* = 5).

The cellular uptake capability of LEVs is crucial for their subsequent bioactivity, as it directly determines their effector capacity and mechanisms of action (16). We therefore evaluated the efficiency of LEV internalization *in vitro* using NCM460 cells (Figure 3A). Following 24-h exposure to varying concentrations of LEVs, cell viability remained high (approximately 95%) at 50 μg/mL, indicating low cytotoxicity (Figure 3B). Hence, this concentration was selected for subsequent experiments. To examine LEV–cell interactions, LEVs were labeled with the lipophilic membrane dye DiO and incubated with NCM460 cells for 0–6 h (Figure 3A). A time-dependent increase in cell-associated green fluorescence was observed after extensive washing, suggesting progressive association with cells and potentially uptake into intracellular compartments. However, because lipophilic dyes may exchange between membranes and can generate background signals, these images should be interpreted as supportive evidence of LEV–cell interaction rather than definitive proof of cytosolic delivery (Figure 3C).

### LEVs attenuated LPS-stimulated inflammatory injury in NCM460 cells and reduced intracellular NO production

**FIGURE 4:**
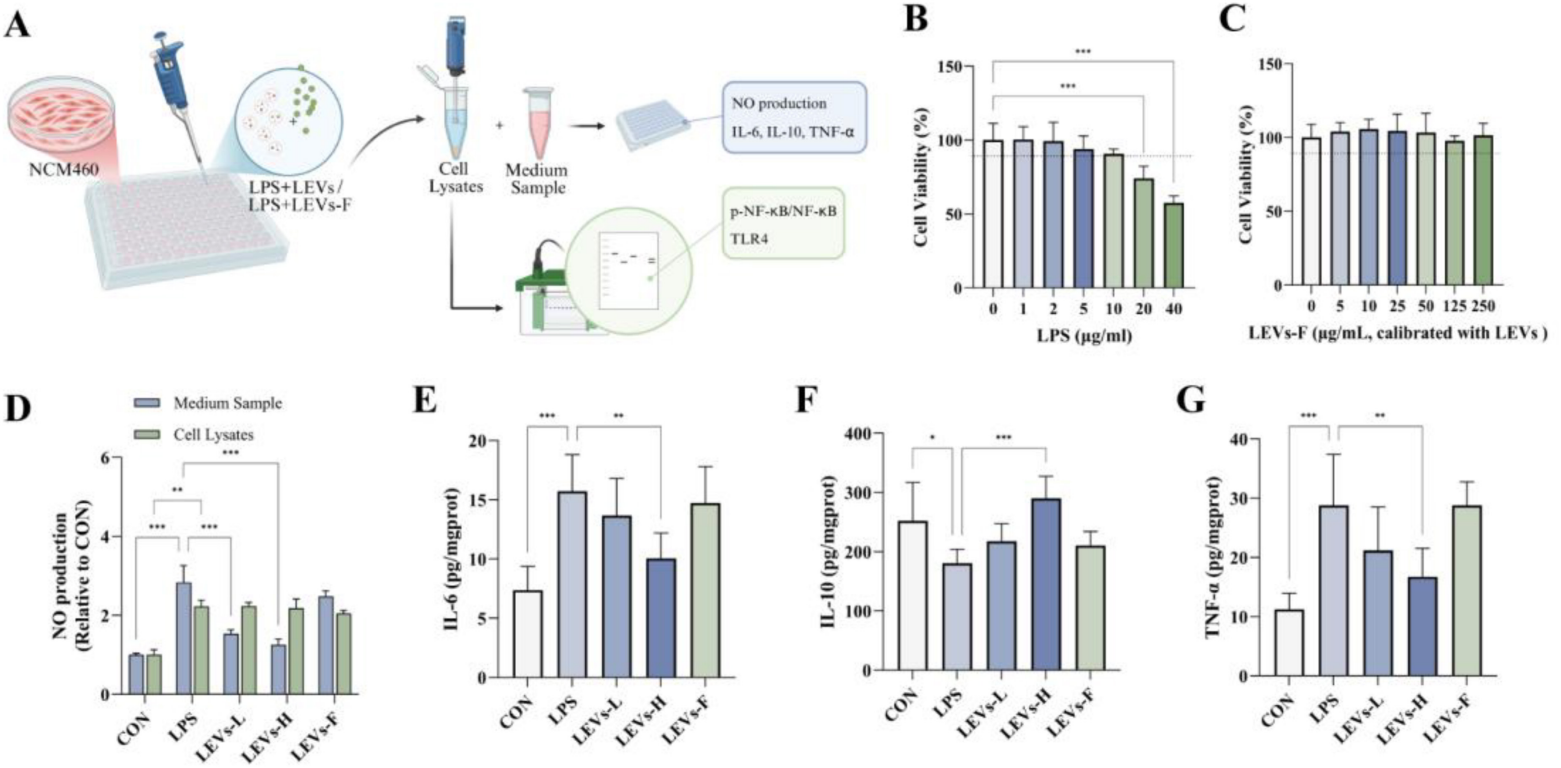
Anti-inflammatory effects of LEVs and LEVs-F in LPS-stimulated NCM460 Cells. **(A)** Experimental design for LEVs or LEVs-F and LPS co-treatment. **(B)** Cell viability assessment following 12 h exposure to LPS (0–40 μg/mL, *n* = 6). **(C)** Cell viability assessment following 24 h exposure to LEVs-F (0–250 μg/mL, *n* = 6). **(D)** Measurement of intracellular and extracellular NO (*n* = 6). **(E–G)** Levels of IL-6, IL-10, and TNF-α under LEVs intervention (*n* = 6). Data were expressed as mean ± SD, *P < 0.05, **P < 0.01 and ***P < 0.001.

The observed cellular uptake of LEVs in NCM460 cells supports their ability to interact with intestinal epithelial cells *in vitro*, providing a basis for subsequent functional assays in this LPS-stimulated epithelial inflammation model. Therefore, we used an LPS-stimulated NCM460 epithelial inflammation model to evaluate whether LEVs modulate oxidative stress–inflammatory responses (Figure 4A). LPS, a well-established inflammatory stimulus, exerts its effects through multiple mechanisms, including the activation of inflammatory pathways and factors, as well as the induction of oxidative stress (17–19). In an LPS-stimulated inflammatory model using NCM460 cells, a 12-h treatment with 10 μg/mL LPS reduced cell survival but did not cause significant cytotoxicity (Figure 4B). Based on the LPS CCK-8 results and previous reports, we selected 10 μg/mL LPS to stimulate an inflammatory response and tested LEVs at two concentrations: 25 μg/mL (LEVs-L) and 50 μg/mL (LEVs-H). In addition, to control for potential contributions from co-isolated soluble components, we prepared an EV-depleted fraction from the post-ultracentrifugation supernatant (LEVs-F) and tested it at a LEVs-H–equivalent dose, at which LEVs-F proved to have no significant cytotoxicity (Figure 4C). Cells were pretreated with LEVs or LEVs-F for 12 h prior to the addition of LPS, which was then co-administered for another 12 h. As shown in Figures 4D–G, LEVs pretreatment reduced intracellular NO production in a dose-dependent manner, whereas NO levels in the culture supernatant were not significantly altered. Notably, LEVs-F did not reduce intracellular NO compared with the LPS group, indicating that the inhibitory effect was primarily associated with the vesicular fraction. Consistently, LEVs significantly decreased IL-6 and TNF-α and increased IL-10, while LEVs-F showed no significant modulation of these cytokines relative to LPS, further supporting that the anti-inflammatory activity was mainly mediated by LEV-associated components.

### LEVs exhibit anti-inflammatory activity in LPS-stimulated cells

**FIGURE 5:**
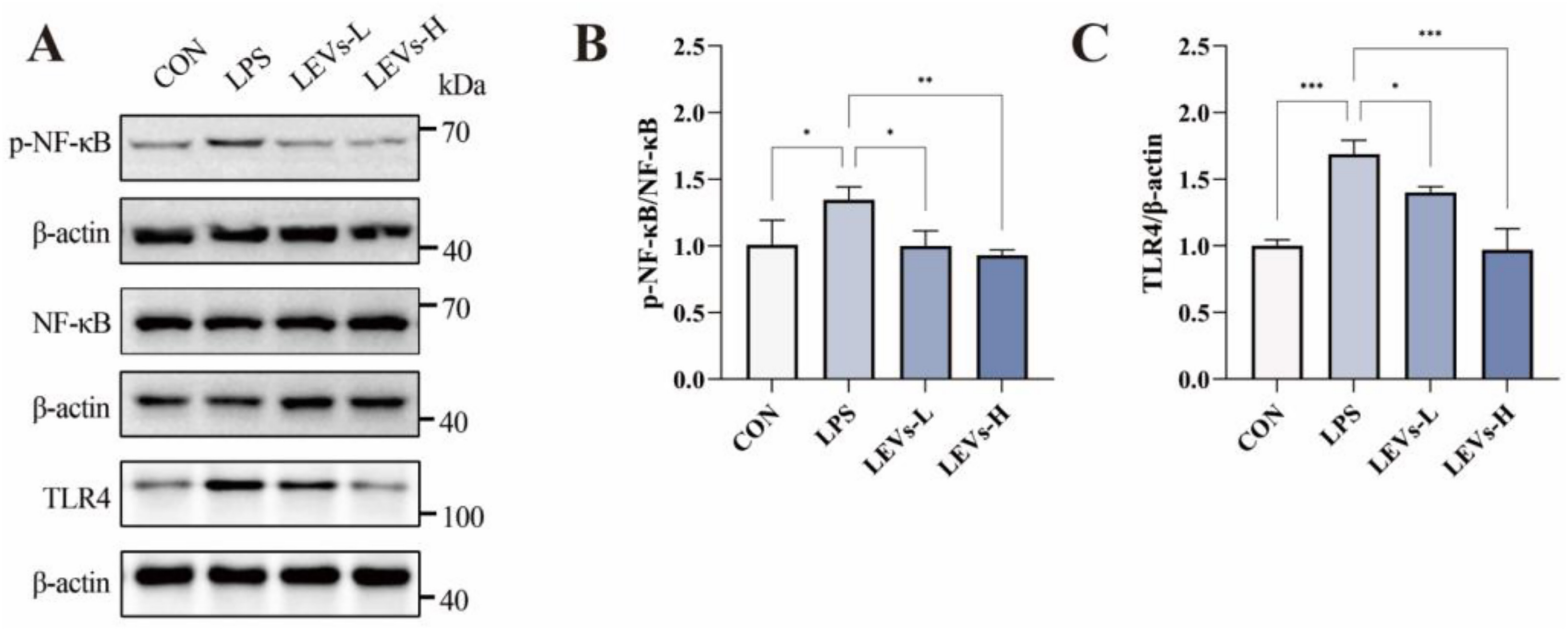
LQYY formula contains EVs (LEVs) downregulated the expression of inflammation-related pathway proteins. **(A–C)** Expression levels of phospho-NF-κB, total NF-κB, and TLR4 (*n* = 3). Data were expressed as mean ± SD, *P < 0.05, **P < 0.01 and ***P < 0.001.

Lipopolysaccharide is well established as a potent inducer of proinflammatory responses. To assess the inflammatory status in LPS-stimulated NCM460 cells, we measured the expression levels of key cytokines. LPS challenge significantly increased the secretion of proinflammatory cytokines IL-6 and TNF-α, while simultaneously suppressing the anti-inflammatory cytokine IL-10. Treatment with a high dose of LEVs (LEVs-H) markedly reversed these aberrant cytokine profiles; however, a low dose (LEVs-L) failed to elicit significant changes, indicating that the anti-inflammatory effect of LEVs is dose-dependent (Figures 4D–F). We further investigated the activation of canonical inflammatory signaling pathways by examining the expression of NF-κB and TLR4. LPS induction significantly upregulated both p-NF-κB and TLR4 protein levels, indicating robust activation of inflammatory signaling. Subsequent treatment with LEVs, particularly LEVs-H, substantially downregulated the expression of both p-NF-κB and TLR4. Consistent with cytokine data, LEVs-H exerted a stronger inhibitory effect on these inflammatory mediators than LEVs-L, further supporting the dose-responsive efficacy of LEVs in mitigating LPS-triggered inflammation (Figures 5A–C).

## Discussion

In this study, we successfully isolated EVs from the LQYY formula and confirmed that LEVs contain abundant bioactive metabolites–including phenylpropanoids, flavonoids, and fatty acids. Furthermore, LEVs were effectively internalized by NCM460 cells and exerted dose-dependent anti-inflammatory effects, as evidenced by specifically inhibited intracellular NO production, regulation of dysregulated inflammatory cytokines, and downregulation of key inflammatory pathway proteins such as NF-κB and TLR4. These findings suggest that LEVs may serve as critical functional mediators of the LQYY formula, alleviating abnormal inflammatory responses via a more direct and efficient delivery mechanism. This interpretation is consistent with the growing view that plant-derived extracellular vesicles can function as next-generation delivery platforms that protect labile bioactive cargo and enhance its bioavailability at mucosal sites (20).

Extracellular vesicles isolated from medicinal plants and related TCMs preparations have increasingly been recognized as bioactive nanocarriers. For example, ginseng-derived EVs-like nanovesicles were reported to protect against diet- and alcohol-associated liver injury while restoring intestinal junctional proteins and rebalancing the gut microbiome after oral administration (21), and *Pueraria lobata*-derived EVs -like nanovesicles were shown to alleviate rheumatoid arthritis by modulating gut microbiota–derived metabolite production (22). These plant-derived EVs demonstrate considerable stability and efficient delivery capabilities under physiological conditions, bypassing conventional secretory and absorptive pathways (23, 24). Recent reviews further emphasize that plant-derived vesicles can be engineered or cargo-enriched to deliver small molecules and nucleic acids with improved safety profiles, supporting their development as orally applicable nanocarriers (20). Their favorable physicochemical properties–including small size, negative surface charge, lipid bilayer membrane, and hydrophilic surface–endow them with excellent mucus permeability, mucosal adhesion, gastrointestinal stability, biocompatibility, and inherent targeting specificity (25, 26). These attributes are particularly valuable for delivering hydrophobic or structurally labile active compounds derived from TCMs, especially since EVs naturally encapsulate bioactive constituents inherent to their botanical origin. For instance, ginger-derived EVs enriched with 6-gingerol and 6-shogaol within their lipid membranes have demonstrated dose-dependent therapeutic effects in gastrointestinal injury models (27). Similarly, EVs from *Pueraria lobata* have been shown to promote M2 macrophage polarization and modulate immune responses, serving as an effective delivery vehicle for puerarin (28). However, research on EVs derived from multi-component TCMs formulations remains limited. We propose that complex compound formulations may exert their effects through EVs in a more intricate manner compared to single herbs. In this study, we aim to explore the role of EVs derived from the LQYY formula to elucidate their potential multifaceted mechanisms of action.

The LQYY formula incorporates multiple lipid-rich medicinal herbs, and our untargeted metabolomics identified several fatty acids among the metabolites detected in the DGUC-purified LEV preparation. Prior studies have reported anti-inflammatory and immunomodulatory activities of some of these fatty acids in other experimental contexts. Palmitoleic acid enhances insulin sensitivity and reduces hepatic lipid accumulation, while *in vitro* studies confirm its ability to attenuate LPS-stimulated macrophage inflammation via inflammasome and NF-κB pathways (29–32). Similarly, lauric acid modulates intestinal immune homeostasis through IgA production regulation (33). Furthermore, LEVs harbor substantial quantities of secondary metabolites involved in phenylpropanoid and flavonoid biosynthesis pathways. Phenylpropanoids exhibit potent antioxidant and anti-inflammatory properties, ameliorating intestinal inflammation and dysbiosis linked to metabolic disorders (34). For instance, salvianolic acid B mitigates intestinal ischemia-reperfusion injury and colitis by reducing oxidative stress and enhancing barrier function (35, 36). Flavonoids–including quercetin, kaempferol, and luteolin–demonstrate antioxidant, anti-inflammatory, and antibacterial effects while stabilizing cellular ion homeostasis (37–40). Notably, quercetin improves intestinal health in colitis models by elevating catalase and superoxide dismutase activities while suppressing IL-17A and IL-22 (41). Extracts rich in these flavonoids (*Serpylli herba*) additionally downregulate cyclooxygenase-2 expression, reducing colonic inflammation (42). In line with this broader concept, EVs-associated lipid metabolites have also been implicated in various hyperinflammation; for example, untargeted profiling of serum EVs-like nanovesicles from COVID-19 patients reported increased levels of several anti-inflammatory metabolites, like LysoPS and 15-d-PGJ2, and proposed a role for EVs-like nanovesicles cargo in modulating inflammatory dysregulation (43). Collectively, these studies all imply the possible positive effects on the gut brought about by natural products enriched in LEVs, and importantly these effects may be further amplified by the structural advantage of EVs. However, we did not directly test purified palmitoleic acid or lauric acid, nor did we perform lipid-fractionation or depletion approaches in our NCM460 model; therefore, these molecules are discussed here as plausible contributors rather than confirmed mediators of the observed effects. Notably, fatty acids are common constituents of plant-derived EVs, raising the possibility that part of the bioactivity reported for different TCMs-EVs preparations may converge on shared metabolite features.

Given that orally administered LEVs primarily interact with the gastrointestinal system, we focused on their effects on colonic epithelial cells. This prioritization stems from their inherent phospholipid bilayer structure, which enables rapid fusion with target membranes rather than requiring transport mechanisms typical of single compounds. In NCM460 cells, LEVs demonstrated exceptional internalization efficiency–a prerequisite for their bioactivity. As previously noted, the lipid bilayer architecture of LEVs confers not only structural stability but also enhanced barrier penetration and biocompatibility (25, 44). Within hours of exposure, LEVs were internalized into the cytoplasm of NCM460 cells, with occasional nuclear localization observed. This rapid intracellular delivery system facilitates direct action of LEVs bioactive cargo–including metabolites and signaling molecules–representing a distinct mechanism compared to conventional compound delivery systems.

Further, we established an LPS-stimulated inflammatory model in NCM460 cells to evaluate the anti-inflammatory and antioxidant properties of LEVs (17). NO, a key redox mediator produced by virtually all human cell types, regulates critical biological processes including immune responses, platelet aggregation, and neurotransmission (45). Elevated NO levels–frequently observed in pathological conditions–are associated with increased oxidative stress (46, 47). In gastrointestinal cells, NO accumulation promotes the formation of highly destructive peroxynitrite, inducing cellular damage (48, 49). In clinical inflammatory bowel diseases (Crohn’s disease and ulcerative colitis), persistent inflammation driven by aberrant immune responses to environmental and microbial factors manifests oxidative stress as a core pathological feature (50). Excessive ROS production impairs intestinal epithelial barrier integrity, increases mucosal permeability, and facilitates luminal antigen/pathogen translocation (51, 52). This oxidative damage sensitization further exacerbates barrier dysfunction, establishing a vicious cycle of chronic inflammation through sustained tissue damage (53). Crucially, NF-κB pathway activation is central to inflammatory progression, where LPS-stimulated TLR-mediated pattern recognition synergizes with oxidative stress to upregulate pro-inflammatory cytokines (IL-6, TNF-α) and chemokines, ultimately compromising epithelial barrier function (54–57). Although these mechanisms are essential for pathogen defense, persistent NF-κB-mediated immune cell recruitment perpetuates chronic inflammation (58, 59). Administration of LEVs significantly mitigated LPS-triggered inflammatory responses in NCM460 cells, attenuating oxidative stress, normalizing inflammatory factors, and modulating pathway activation. These findings collectively demonstrate that LEVs confer anti-inflammatory and antioxidant activities in intestinal epithelial cells.

In this study, we investigated EVs as potential active components of the LQYY formula, hypothesizing that they might exert inhibitory effects on oxidative stress and inflammatory responses in intestinal cells due to their cargo of diverse natural metabolites. Following the successful isolation and characterization of LEVs from the LQYY formula, we demonstrated that LEVs significantly attenuated LPS-stimulated inflammatory activation in a dose-dependent manner.

Nevertheless, several limitations should be acknowledged. Because LQYY comprises multiple herbs, the DGUC-purified LEVs likely represent a heterogeneous pool originating from different botanical sources and vesicle subtypes, which prevents assignment of specific bioactivities or individual metabolites to a single herb and may contribute to batch-to-batch variability. Although density-gradient purification and inclusion of an EV-depleted fraction control reduce concerns regarding soluble carryover, we cannot definitively distinguish intravesicular cargo from surface-associated or tightly co-purified components without additional fraction-comparison and vesicle-disruption experiments. In addition, the DiO-based assay provides supportive evidence for LEV–cell association and putative internalization, but the absence of a label-only control and the known caveats of lipophilic dyes warrant more rigorous orthogonal labeling and quantitative imaging in future work. Finally, our conclusions are based on an *in vitro* LPS-stimulated epithelial inflammation model; *in vivo* validation within the multicellular intestinal microenvironment and mechanistic deconvolution of candidate cargo molecules will be required to establish disease relevance and causality.

## Conclusion

In summary, LEVs derived from the LQYY formula are natural nanoparticles enriched with diverse bioactive compounds. These LEVs effectively modulate LPS-stimulated oxidative stress and inflammatory responses in intestinal epithelial cells, thereby mitigating cellular damage and promoting mucosal recovery. The efficacy of LEVs is likely attributable to their high concentration of encapsulated active constituents–such as fatty acids, phenylpropanoids, and flavonoids–coupled with their unique structural properties that enhance bioavailability and target specificity compared to the direct administration of crude extracts. These findings offer mechanistic insight into how formula-derived vesicles may contribute to bioactivity in an epithelial inflammation setting. While suggestive of potential relevance to gastrointestinal inflammation, our results are limited to an *in vitro* model and should be considered hypothesis-generating; further validation in animal models is required.

## Data Availability

The original contributions presented in this study are included in this article/supplementary material, further inquiries can be directed to the corresponding authors.
